# Toward Imaging of the GABA Transporter Type 1 In Vivo: Quantitative Evaluation of 4 Novel PET Radiotracers in Nonhuman Primates

**DOI:** 10.2967/jnumed.125.270332

**Published:** 2025-10

**Authors:** Paul Gravel, Jiwei Gu, Chao Wang, Tommaso Volpi, Jean-Dominique Gallezot, Daniel Holden, Krista Fowles, Ming-Qiang Zheng, Li Zhang, Edilio Borroni, Michael Honer, Luca Gobbi, Gilles Tamagnan, Yiyun Huang, Richard E. Carson

**Affiliations:** 1 PET Center, Department of Radiology and Biomedical Imaging, Yale University, New Haven, Connecticut;; 2 Pharma Research and Early Development, Roche Innovation Center Basel, F. Hoffmann-La Roche Ltd., Basel, Switzerland; and; 3 Department of Psychiatry, Yale University, New Haven, Connecticut

**Keywords:** GABA, GAT-1, PET imaging, kinetic modeling

## Abstract

The main inhibitory neurotransmitter in the central nervous system is γ-aminobutyric acid (GABA). GABA transporter type 1 (GAT-1) is the principal GABA transporter in the brain, and it plays a crucial role in modulating GABA signaling. Its potential role in several neuropsychiatric disorders makes it an important target to study. Although PET radiotracers exist for the GABA receptors, none have been successful for imaging GAT-1. The focus of this work was to evaluate the kinetic behavior of 4 novel ^18^F-labeled PET radiotracers (^18^F-GATT-31, ^18^F-GATT-34, ^18^F-GATT-39, and ^18^F-GATT-44) for imaging GAT-1 in nonhuman primates and to select the best radiotracer to progress to human studies. **Methods:** Twenty scans were acquired from 4 rhesus monkeys (*Macaca mulatta*). Each monkey received 0.5 mg/kg of tiagabine given approximately 20 min before radiotracer injection and underwent baseline and blocking scans with ^18^F-GATT-31, ^18^F-GATT-34, ^18^F-GATT-39, or ^18^F-GATT-44 on a small-animal PET scanner. During each scan, arterial blood was collected for measurement of the input function. Kinetic analysis was performed using a 1-tissue compartment model, 2-tissue reversible model (*k*_4_ > 0), and 2-tissue irreversible model (*k*_4_ = 0), including a blood volume fraction term and a time-delay term. **Results:** All radiotracers exhibited good, albeit slow, brain uptake within the cortical and subcortical gray matter regions and cerebellum. Peripheral metabolism was slow for ^18^F-GATT-34, ^18^F-GATT-39, and ^18^F-GATT-44, with greater than 75% remaining as the parent compound, but was somewhat faster for ^18^F-GATT-31 (63%) over the 3-h scans. The 1-tissue compartment model delivered a reliable performance on the basis of the overall lowest Akaike information criterion and an SE of less than 10% for the volume of distribution. ^18^F-GATT-39 and ^18^F-GATT-34 were eliminated from progressing to human studies because of low brain uptake or low specific binding. The 2 remaining radiotracers had similar characteristics, with ^18^F-GATT-44 showing slightly superior performance over ^18^F-GATT-31, with more consistent tiagabine blocking results (65%–71%) and with nondisplaceable binding potential (BP_ND_) values ranging from 1.2 to 4.2 across gray matter structures. **Conclusion:** We successfully developed 4 GAT-1 selective radiotracers and evaluated them in nonhuman primates with kinetic analysis and blocking studies with tiagabine. Of these compounds, ^18^F-GATT-44 exhibited consistent results and reasonable BP_ND_ values and will progress to human studies.

## Visual Abstract

**Figure:**
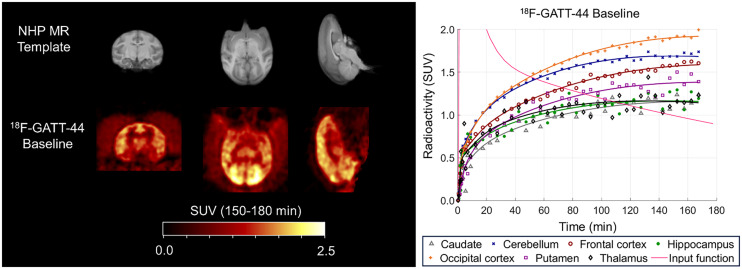

The main inhibitory neurotransmitter in the central nervous system is γ-aminobutyric acid (GABA). GABAergic neurons represent more than 20% of cortical neurons (1). GABA is released in the synaptic cleft via exocytosis and binds to GABA subtype A (GABA_A_) and subtype B receptors, from which an inhibitory signal is sent to the postsynaptic neuron. The effect of GABA is terminated by its reuptake from the synaptic cleft through 1 of 4 GABA transporter (GAT) isoforms, belonging to the solute carrier family SLC-6: GAT transporter type 1 (GAT-1), type 2, type 3, and betaine–GABA transporter 1 (1,2). The blockade of GATs, and thus GABA reuptake, produces anticonvulsant properties (3). Tiagabine, a drug approved by the Food and Drug Administration, has been developed as a GABA reuptake inhibitor and is used clinically for the treatment of epilepsy (4,5). Tiagabine has excellent selectivity for GAT-1, as demonstrated in vitro in the human brain postmortem (5). It has also been shown to increase GABA in the vervet brain in vivo (6). GAT-1 is the most abundant GABA transporter of the 4 GAT subtypes in the mammalian brain, mainly located in presynaptic neuronal terminals and, to some extent, in astrocytic processes, oligodendrocytes, and microglia (7,8). It is dominantly expressed in the cerebral cortex (340 nM), with lower concentrations in striatum (5,7) and white matter (8). Its key role in modulating GABA signaling, and thus the brain’s excitatory–inhibitory balance, and its potential implication in several neuropsychiatric disorders, such as epilepsy (9), schizophrenia (10), anxiety (11), depression (12), Alzheimer disease (13), and neuroinflammation (14), makes it an important target to study.

PET radiotracers for GABA receptors have been developed, such as [^11^C]flumazenil and [^11^C]Ro15-4513, which specifically act on GABA_A_ receptors as an antagonist and inverse agonist of its benzodiazepine binding site, respectively. [^11^C]Ro15-4513 has high affinity for the GABA_A_ receptor α5 subtype (15,16). Although these radiotracers provide information on postsynaptic GABA function, no radiotracers have been successfully developed for GAT-1 or other GABA presynaptic targets (16–18). One recent attempt to develop a GAT-1 radiotracer from a tiagabine analog showed brain uptake in the rhesus monkey, but it was too limited to allow quantitative in vivo imaging (18). As highlighted by Andersson et al. (15), major difficulties encountered with the development of GAT-1 radiotracers, initially related to limited understanding of GAT-1 pharmacology and distribution, and concerns about the pharmacologic effects of GAT-1 inhibitors, have been addressed. However, slow brain uptake of hydrophilic tiagabine analogs, which cannot use carrier-mediated transport to cross the blood–brain barrier (BBB), still represents an obstacle to successful development of GAT-1 PET radiotracers (3).

We have developed multiple radiotracers for imaging GAT-1 based on the structure of tiagabine (19,20). These novel radiotracers have high affinity and selectivity for GAT-1, cross the BBB, have good uptake in the brain, and exhibit GAT-1–specific binding that can be blocked with tiagabine and quantified by kinetic analysis. These represent a breakthrough in GAT-1 radiotracer development. Here, we report the detailed evaluation of 4 novel GAT-1 radiotracers in nonhuman primates with quantitative modeling analyses.

## MATERIALS AND METHODS

### Radiochemistry of GAT-1 Radiotracers

Radiosynthesis was performed via copper-mediated nucleophilic fluorination of arylstannane precursors. Briefly, the organostannane precursors were dissolved in *N*,*N*-dimethylacetamide and reacted with [^18^F]KF, copper(II) trifluoromethanesulfonate, and pyridine at 120 °C for 20 min. This step yielded the ester intermediates, which were subsequently hydrolyzed under basic conditions to remove the protecting group, providing the target radioligand in high radiochemical purity and molar activity. A detailed description of the procedure is provided in the supplemental materials, available at http://jnm.snmjournals.org.

### PET Scanning Protocol

**FIGURE 1.**
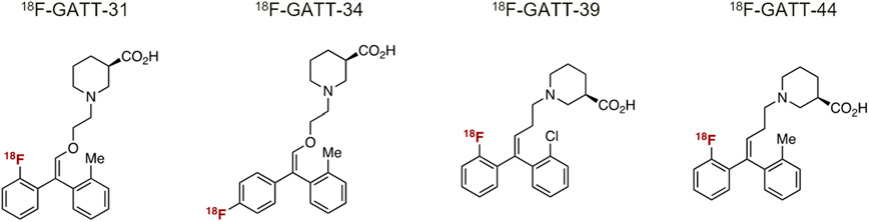
Chemical structures of ^18^F-GATT-31, ^18^F-GATT-34, ^18^F-GATT-39, and ^18^F-GATT-44.

Twenty scans were acquired from 4 rhesus monkeys (*Macaca mulatta*). Each monkey underwent baseline and blocking scans with ^18^F-GATT-31, ^18^F-GATT-34, ^18^F-GATT-39, or ^18^F-GATT-44 (Fig. 1) on a Focus-220 small-animal PET scanner (Siemens Healthineers). The injection parameters are summarized in Table 1.

**TABLE 1.** Injection Parameters and Plasma Analysis for GAT-1 Radiotracers in Nonhuman Primates

Parameter	^18^F-GATT-31 (*n* = 6)	^18^F-GATT-34 (*n* = 4)	^18^F-GATT-39 (*n* = 4)	^18^F-GATT-44 (*n* = 6)
Injected dose (MBq)	170 ± 28	181 ± 6	176 ± 12	182 ± 4
Injected mass (ng/kg)	122 ± 73	60 ± 18	52 ± 26	56 ± 54
Free fraction (%)	12.4 ± 1.8	10.8 ± 0.4	10.5 ± 1.0	13.5 ± 1.4
Molar activity (MBq/nmol)*	66 ± 40	110 ± 34	108 ± 47	189 ± 105
Parent compound (%)				
30 min	87 ± 3	91 ± 2	95 ± 4	88 ± 4
90 min	76 ± 6	83 ± 2	85 ± 9	83 ± 6
180 min	63 ± 11	75 ± 5	81 ± 9	79 ± 7

* Molar activity at end of synthesis.

Data represent mean ± SD.

Before each scan, each monkey was sedated with alfaxalone (2 mg/kg), midazolam (0.3 mg/kg), and dexmedetomidine (0.01 mg/kg), and anesthesia was maintained with 1.5%–2.5% isoflurane. Heart rate, blood pressure, respiration rate, and oxygen saturation were monitored continuously. An intravenous line was placed in the saphenous or cephalic vein for delivery of the radiotracer, as well as tiagabine during blocking studies (0.5 mg/kg administered 19 ± 4 min before radiotracer injections). A catheter was placed in the radial or tibial artery for blood sampling.

All procedures were approved by the Yale University Institutional Animal Care and Use Committee under guidelines consistent with the U.S. Government Principles for the Use and Care of Vertebrate Animals Used in Testing, Research, and Training; the Animal Welfare Act; and Animal Welfare Regulations. Animal data are reported in accordance with the ARRIVE 2.0 guidelines (21).

### PET Image Acquisition and Processing

Before injection, a transmission scan was acquired. Starting with injection of the radiotracer, PET emission data were acquired in list-mode format and binned into 45 frames (6 × 30 s, 3 × 1 min, 2 × 2 min, and 34 × 5 min). These data were reconstructed with a Fourier rebinning/filtered back-projection algorithm with corrections for attenuation, scatter, and randoms.

For region-of-interest (ROI) definition, each PET image was registered to an in-house rhesus monkey anatomical MRI template using the concatenation of a rigid-body transformation between the summed PET image (0–10 min) and corresponding monkey’s MRI (using the FMRIB Linear Image Registration Tool) and a nonlinear transformation matrix estimated between the monkey’s MRI and the MRI template (BioImage Suite software version 3.01). Regional time–activity curves were extracted for the following 11 ROIs: brain stem, caudate, cerebellum, cingulate cortex, frontal cortex, hippocampus, insula, occipital cortex, putamen, temporal cortex, and thalamus.

### Arterial Input Function

Arterial blood samples were collected for each scan to measure the input function. Metabolite analysis was performed using an automatic column-switching high-performance liquid chromatography system (22) for 7 arterial blood samples collected at 5, 15, 30, 60, 90, 120, and 180 min. The radiometabolite-corrected arterial plasma input function was obtained by calculating the product of the fitted total plasma (fitted with a sum of exponentials) and unmetabolized parent fraction curves (using an integrated γ-function (23)).

The plasma free fraction (*f*_p_) for each scan was measured in triplicate using the ultrafiltration method and calculated as the ratio of radioactivity in the filtrate to that in the plasma (24).

### Plasma Tiagabine Concentration

For 8 of the 10 blocking scans, venous blood samples were acquired at 30, 60, 90, 120, 160, and 190 min after injection of tiagabine, in addition to 1 control sample before tiagabine injection, to measure its concentration using mass spectrometry (described in the supplemental materials).

### Kinetic Modeling of GAT-1 Radiotracers

For each baseline and blocking scan, kinetic analysis was performed using a 1-tissue compartment model (1TCM), a reversible 2-tissue compartment model (2TCM; *k*_4_ > 0), and an irreversible 2-tissue compartment model (2TiCM; *k*_4_ = 0), including a blood volume fraction (*V*_b_) term and a time-delay term. *V*_b_ was estimated for each time–activity curve using the whole-blood curve fitted with a sum of exponentials. The time delay (Δ*t*) between the input function and the brain was estimated by fitting the first 10 min of the whole-brain time–activity curve to a 1TCM with 3 parameters: *K*_1_, *V*_b_, and Δ*t* (*k_2_* was set to 0 because of the negligible radiotracer washout during the first 10 min). This estimated time delay value was then used in the time–activity curve fits. The primary parameters of interest were the influx constant (*K*_1_, expressed in microliters per minute per cubic centimeter, since values were low), the volume of distribution (*V*_T_, expressed in milliliters per cubic centimeter), and the net influx constant (*K*_i_, expressed in milliliters per minute per cubic centimeter) for 2TiCM.

Selection of the best model was based on the *F*-test, Akaike information criterion (25), and reliability of the primary parameters using the relative SE (rSE).

Occupancy plots (26) were used to estimate tiagabine occupancy and the nondisplaceable volume of distribution (*V*_ND_). This *V*_ND_ value was used to compute the nondisplaceable binding potential (BP_ND_ = *V*_T_/*V*_ND_ − 1). To compare the performance between 2 radiotracers, a Guo plot (27) was created, using baseline *V*_T_ values, to assess the relative affinity and BP_ND_ values of each radiotracer.

GAT-1 radiotracers were deemed promising on the basis of *K*_1_ values (if values were too low, radiotracers were discarded), consistency of *V*_ND_ values across animals, mean BP_ND_ values, and consistency of tiagabine occupancy values across animals.

Finally, to assess the possibility of reducing scan duration, time stability was assessed for the most promising radiotracer by sequentially shortening the time–activity curves from 180 to 60 min and reestimating the kinetic parameters. Scan-time reduction was deemed possible if the *V*_T_ values from these shortened time–activity curves were within 10% of those estimated with the full time–activity curves.

## RESULTS

### Injection Parameters and Parent Compound

Table 1 depicts the injection parameters and blood measurements for the radiotracers. The injected doses (170–182 MBq) and free fraction (10%–14%) were similar for all radiotracers. The injected mass for ^18^F-GATT-31 was about twice that of the other radiotracers. The percent parent compound was quite high and similar for ^18^F-GATT-34, ^18^F-GATT-39, and ^18^F-GATT-44, with 75%–80% of the radiotracers being unmetabolized at 180 min, but was somewhat lower for ^18^F-GATT-31 (63% unmetabolized at 180 min).

### Tiagabine Parameters

The 0.5 mg/kg tiagabine dose produced an average plasma concentration (30–190 min after tiagabine injection) of 385 ± 70 µg/L. This is comparable to the plasma concentration found in humans with a 24-mg single oral dose, which has a peak value of 552 µg/L with an elimination half-life of 7.3 h (4).

### Brain Uptake of GAT-1 Radiotracers

**FIGURE 2.**
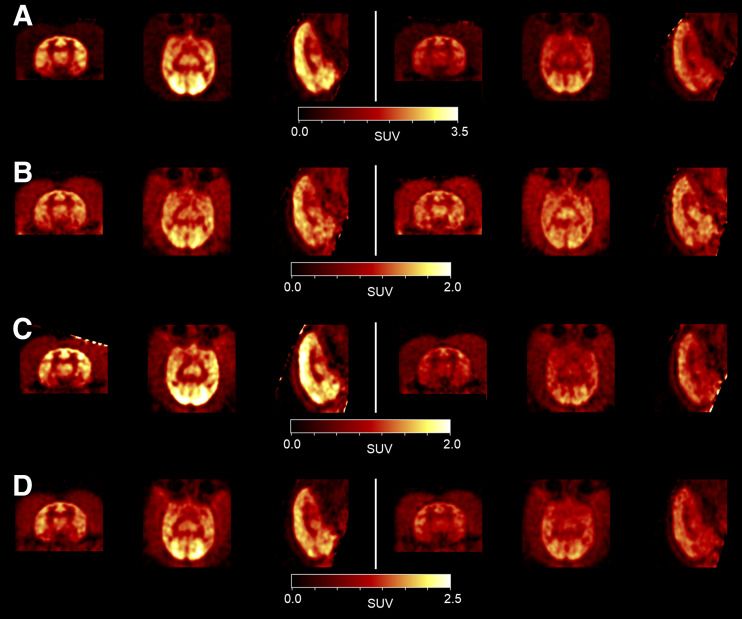
SUV images (150–180 min) for 1 monkey in template space at baseline (left) or blocking (right) after pretreatment with tiagabine (0.5 mg/kg) for ^18^F-GATT-31 (A), ^18^F-GATT-34 (B), ^18^F-GATT-39 (C), and ^18^F-GATT-44 (D). For clarity, color scale differs for each radiotracer.

**FIGURE 3.**
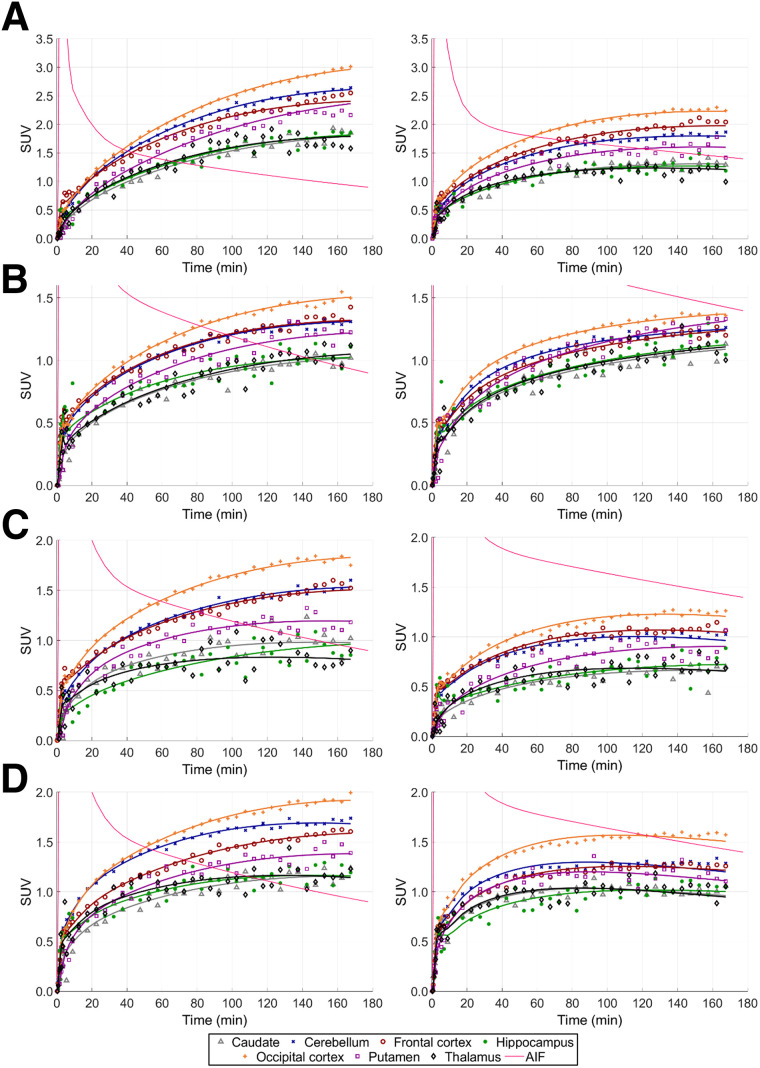
SUV time–activity curves for 1 monkey at baseline (left) and after blocking (right), with corresponding kinetic modeling fits (solid lines) using 1TCM for ^18^F-GATT-31 (A), ^18^F-GATT-34 (B), ^18^F-GATT-39 (C), and ^18^F-GATT-44 (D). For clarity, *y*-axis range differs for each radiotracer. AIF = arterial input function (metabolite corrected).

PET SUV images, summed from 150 to 180 min after injection, are displayed in Figure 2 with corresponding time–activity curves (Fig. 3) for 1 monkey. All radiotracers exhibited good brain uptake, albeit slow, within cortical and subcortical gray matter regions and the cerebellum. SUVs were highest in the occipital and cingulate cortices and lowest in the caudate, hippocampus, and thalamus for all radiotracers, with the rank of the other ROI SUVs slightly varying among radiotracers. The brain time–activity curves, displayed in Figure 3, show slow uptake for all radiotracers, which is still ongoing at 180 min. As shown in Figures 2 and 3, there is a clear reduction in brain uptake between baseline and blocking conditions (except for ^18^F-GATT-34), whereas the plasma activity increased in the blocking study. The time–activity curves (Fig. 3) generally depict a narrower inter-ROI spread among the blocking scans compared with the baseline scans, consistent with a reduction in specific binding.

### Kinetic Modeling of GAT-1 Radiotracers

Of 220 time–activity curves (20 scans × 11 ROIs), only 19 had a significantly better fit (*F*-test; *P* ≤ 0.05 and lowest Akaike information criterion) with the 2TiCM and 72 with the 2TCM, compared with the 1TCM. Of those, only 5 and 47 time–activity curves had a *K*_i_/*V*_T_ rSE of no more than 10% for the 2TiCM and 2TCM, respectively. On the other hand, the 1TCM delivered better fits for 129 ROIs, in addition to having 186 ROIs with reliable *V*_T_ rSE of no more than 10%. Furthermore, we compared the *V*_T_ values between 1TCM and 2TCM when the rSE was less than 10% for both models. *V*_T_ values for the 1TCM and the 2TCM were significantly correlated (*R* = 0.987), with the 1TCM yielding values approximately 10% lower for larger *V*_T_ values (Supplemental Fig. 1). Therefore, given the more reliable estimates and the consistency with the 2TCM, we chose the 1TCM as the best model. Figure 3 shows the time–activity curve fits for the 1TCM.

**FIGURE 4.**
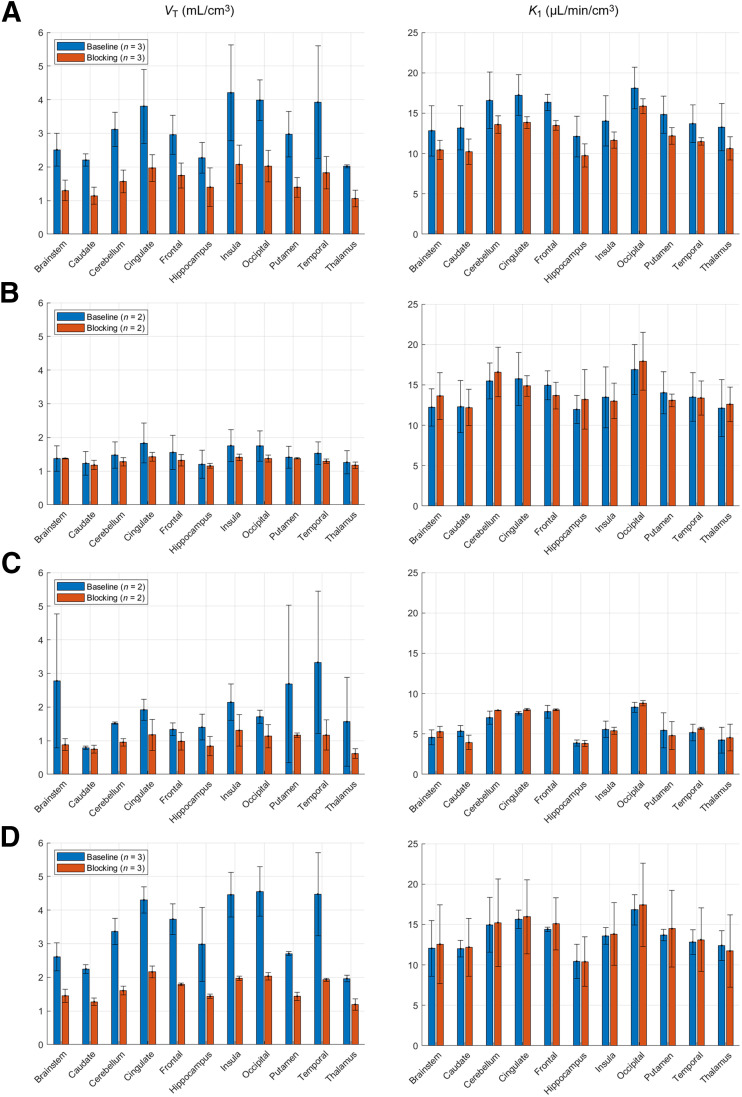
Bar plots of mean and SD of *V*_T_ (left) and *K*_1_ (right) estimates for each radiotracer, averaged across monkeys for baseline and blocking studies for ^18^F-GATT-31 (A), ^18^F-GATT-34 (B), ^18^F-GATT-39 (C), and ^18^F-GATT-44 (D). Units for *V*_T_ are mL/cm^3^, and units for *K*_1_ are μL/min/cm^3^.

Figure 4 displays the regional 1TCM *V*_T_ and *K*_1_ values for each radiotracer per condition. The median baseline and blocking *V*_T_ values across ROIs were as follows: for ^18^F-GATT-31 (*n* = 3), 2.8 mL/cm^3^ (range, 1.9–5.8 mL/cm^3^) at baseline and 1.5 mL/cm^3^ (range, 0.8–2.5 mL/cm^3^) after blocking; for ^18^F-GATT-34 (*n* = 2), 1.5 mL/cm^3^ (0.9–2.2 mL/cm^3^) at baseline and 1.3 mL/cm^3^ (range, 1.1–1.5 mL/cm^3^) after blocking; for ^18^F-GATT-39 (*n* = 2), 1.6 mL/cm^3^ (0.6–4.8 mL/cm^3^) at baseline and 0.9 mL/cm^3^ (range, 0.5–1.6 mL/cm^3^) after blocking; and for ^18^F-GATT-44 (*n* = 3), 3.5 mL/cm^3^ (1.9–5.9 mL/cm^3^) at baseline and 1.7 mL/cm^3^ (1.0–2.4 mL/cm^3^) after blocking. Overall, baseline values were highest in the cingulate, occipital, temporal, and insular cortices and in the lower range for the thalamus and caudate nucleus. *V*_T_ values were highly variable for ^18^F-GATT-39. In addition, there were radiotracer-specific differences in between-animal global *V*_T_ values (ROI average) expressed as percent SD at baseline and after blocking: 21% and 22% for ^18^F-GATT-31, 27% and 7% for ^18^F-GATT-34, 38% and 26% for ^18^F-GATT-39, and 13% and 4% for ^18^F-GATT-44.

*K*_1_ values were well-estimated for all radiotracers, with an rSE of less than 10% for all but 2 ROIs (^18^F-GATT-39). No change in *K*_1_ values was observed between baseline and blocking conditions.

^18^F-GATT-39 had especially low *K*_1_ values (Fig. 4C). Furthermore, the rSE values for ^18^F-GATT-39 *V*_T_ were large (23% ± 35%), compared with 6% ± 4% for ^18^F-GATT-31, 3% ± 2% for ^18^F-GATT-34, and 6% ± 4% for ^18^F-GATT-44. Therefore, this radiotracer was removed from further consideration.

### Blocking Studies

**FIGURE 5.**
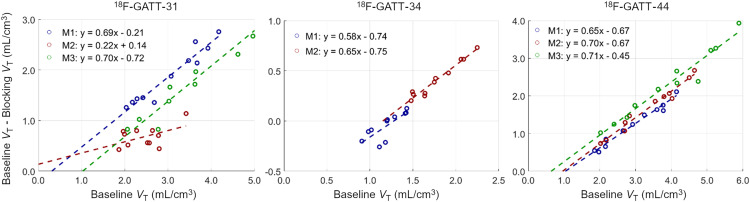
Occupancy plots using *V*_T_ values for ^18^F-GATT-31, ^18^F-GATT-34, and ^18^F-GATT-44, using GAT-1 inhibitor tiagabine. For clarity, axis ranges differ for each radiotracer.

Figure 5 displays the occupancy plots for ^18^F-GATT-31, ^18^F-GATT-34, and ^18^F-GATT-44. In most cases, occupancy values across radiotracers and animals were consistent (range, 58%–71%). For ^18^F-GATT-34, monkey 1 had some blocking *V*_T_ values (5/11 ROIs) that were higher than baseline values. Overall, the blocking effect for this radiotracer was small (Fig. 4B). Therefore, ^18^F-GATT-34 was also excluded from further consideration.

The occupancy results for ^18^F-GATT-31 and ^18^F-GATT-44 are summarized in Table 2. For ^18^F-GATT-31, the results for monkey 2 were inconsistent, with an occupancy of only 22% and an unrealistic *V*_ND_ of −0.61 mL/cm^3^. In addition, the *V*_ND_ of 0.31 mL/cm^3^ for monkey 1 had a large rSE (53%). On the other hand, ^18^F-GATT-44 delivered a consistent occupancy of 65%–71% for all monkeys, with *V*_ND_ values ranging from 0.64 to 1.04 mL/cm^3^ (0.88 ± 0.21 mL/cm^3^; average rSE of 16%). Thus, based on the occupancy data, ^18^F-GATT-44 appeared to be more promising than ^18^F-GATT-31.

**TABLE 2.** Summary of Occupancy-Related Parameters for ^18^F-GATT-31 and ^18^F-GATT-44

	^18^F-GATT-31			^18^F-GATT-44		
Parameter	M1	M2	M3	M1	M2	M3
Occupancy (%)	69	22	70	65	70	71
*V*_ND_ (mL/cm^3^)	0.31	−0.61*	1.02	1.03	0.96	0.64

* Unrealistic value.

M1 = monkey 1; M2 = monkey 2; M3 = monkey 3.

To confirm the suitability of ^18^F-GATT-44, a Guo plot comparing ^18^F-GATT-44 and ^18^F-GATT-31 was created (Supplemental Fig. 2). The linearity of the plot indicates that ^18^F-GATT-31 and ^18^F-GATT-44 bind to the same target. Since the *y*-intercept (1 − BP_ND_^GATT-44^/BP_ND_^GATT-31^) was slightly negative, ^18^F-GATT-44 BP_ND_ values are predicted to be somewhat higher (by approximately 17%) than those for ^18^F-GATT-31. Finally, the slope (*f*_p_^GATT-44^*K*_D_^GATT-31^/[*f*_p_^GATT-31^*K*_D_^GATT-44^]) provides estimates of the affinity ratio. Using the measured *f*_p_ values (Table 1), the *K*_D_ ratio (*K*_D_^GATT-31^/*K*_D_^GATT-44^) was 1.06. Therefore, ^18^F-GATT-44 is predicted to have an affinity slightly higher than that of ^18^F-GATT-31.

Based on these results, ^18^F-GATT-44 delivered more consistent overall results and is a slightly superior radiotracer than ^18^F-GATT-31. Thus, ^18^F-GATT-44 will progress to human studies. The regional *V*_T_ and corresponding BP_ND_ values are depicted in Table 3.

**TABLE 3.** Regional *V*_T_ and BP_ND_ Values, Averaged Across 3 Monkeys, for ^18^F-GATT-44

	*V*_T_ (mL/cm^3^)		BP_ND_*	
ROI	Baseline	Blocking	Baseline	Blocking
Brain stem	2.61 ± 0.42	1.45 ± 0.20	1.98 ± 0.48	0.66 ± 0.23
Caudate nucleus	2.25 ± 0.13	1.27 ± 0.11	1.57 ± 0.15	0.45 ± 0.12
Cerebellum	3.36 ± 0.39	1.60 ± 0.13	2.84 ± 0.44	0.83 ± 0.15
Cingulate cortex	4.30 ± 0.39	2.16 ± 0.18	3.91 ± 0.44	1.46 ± 0.20
Frontal cortex	3.73 ± 0.46	1.79 ± 0.04	3.26 ± 0.52	1.04 ± 0.04
Hippocampus	2.98 ± 1.10	1.44 ± 0.07	2.40 ± 1.25	0.64 ± 0.08
Insula	4.46 ± 0.67	1.97 ± 0.07	4.08 ± 0.76	1.25 ± 0.08
Occipital cortex	4.55 ± 0.74	2.04 ± 0.11	4.19 ± 0.84	1.32 ± 0.13
Putamen	2.71 ± 0.05	1.44 ± 0.12	2.09 ± 0.06	0.64 ± 0.14
Temporal cortex	4.48 ± 1.24	1.93 ± 0.05	4.10 ± 1.41	1.20 ± 0.05
Thalamus	1.96 ± 0.10	1.19 ± 0.17	1.24 ± 0.11	0.36 ± 0.20
Average	3.40 ± 0.52	1.66 ± 0.11	2.88 ± 0.59	0.90 ± 0.13

* BP_ND_ = *V*_T_ / *V*_ND_ – 1, using averaged *V*_ND_ values (^18^F-GATT-44), shown in Table 2.

Data represent mean ± SD.

### Time Stability

Supplemental Figure 3 displays the time stability results for ^18^F-GATT-44 at baseline. By shortening the scan from 180 to 120 min, the mean percent differences, calculated across the 3 monkeys, in *V*_T_ values range from −12% (thalamus) to 1% (hippocampus), with interanimal SDs of approximately 11%. Thus, only minimal shortening of the scan is feasible. These results are consistent with the kinetics of ^18^F-GATT-44. Time stability was better for the blocking study. The corresponding mean percent differences with respect to the 180-min value were lower for the blocking condition, with a range of −5% (frontal cortex) to 3% (thalamus), with an SD of approximately 7%. This result was expected because the time–activity curves reached equilibrium faster.

## DISCUSSION

In this work, we compared 4 potential GAT-1 radiotracers in nonhuman primates to select the best radiotracer to progress to human studies and to determine the most appropriate kinetic model. All radiotracers exhibited brain penetration, albeit with slow uptake (Fig. 3). To choose the best radiotracer, we evaluated brain kinetics and specific blocking. Two of the radiotracers were eliminated (^18^F-GATT-39 and ^18^F-GATT-34) because of low brain uptake or low specific binding. The remaining 2 compounds had similar characteristics, with ^18^F-GATT-44 showing slightly superior performance compared with ^18^F-GATT-31, with more consistent results (Table 2). ^18^F-GATT-44 delivered reasonable regional BP_ND_ values (Table 3), ranging from 1.24 to 4.19 at baseline, with the higher values in the cortical regions and lower values in the subcortical regions, as previously observed with ^3^H-tiagabine (5).

The shape of the time–activity curves (Fig. 3) could give the impression that radiotracer uptake is irreversible. However, with the low *K*_1_ values, this is not the case, and the reversible 1TCM was chosen as the most appropriate model. One consequence of low *K*_1_ values is that the early phase of the time–activity curve is considerably impacted by vascular activity, so it is important to include *V*_b_ in the modeling (mean ± SD for ^18^F-GATT-44, 0.036 ± 0.013 mL/cm^3^; *n* = 66, i.e., 11 ROIs × 6 scans).

The slow uptake of radiotracers in the brain is noteworthy. Despite the zwitterionic nature of the nipecotic acid moiety in tiagabine and its derivatives, the passive parallel artificial membrane permeability assay indicates good permeability (17), suggesting that these compounds should theoretically cross the BBB without significant hindrance. Active efflux at the BBB may offer some explanation. Although only marginal efflux by P-glycoprotein (PgP) was measured—with efflux ratios ranging from 1.9 to 2.4 (17)—this may already be sufficient to impede BBB crossing when the compounds are administered at the microdose levels used in PET imaging. Notably, ^18^F-GATT-31 exhibits low efflux by PgP, and the other 3 radiotracers are categorized as nonsubstrates for PgP. It is important to consider that PgP is only one of several mechanisms responsible for efflux at the BBB; other transporters may play a role in these observations.

Of note, the regional distribution of ^18^F-GATT-44 in nonhuman primates, with high values in the cortex and lower values in subcortical regions, is consistent with that of ^11^C-flumazenil, which binds allosterically to GABA_A_ receptors. The overall spatial correlation was calculated as *r* = 0.87 (*P* < 0.01), as determined from previously acquired data (Supplemental Fig. 4). This suggests a substantial degree of colocalization between the presynaptic GABA reuptake machinery (GAT-1) and postsynaptic GABA_A_ receptors. Nevertheless, as more PET experiments are performed with ^18^F-GATT-44, we expect that GAT-1 and GABA_A_ imaging will provide both differential and complementary information in the same manner that has been found in the dopaminergic and serotonergic systems. Furthermore, imaging of the GABA system would be further enhanced by the development of radiotracers targeting the GABA subtype B receptor and the orthosteric site of the GABA_A_ receptor, for which no radiotracers exist to date.

The next step is to progress ^18^F-GATT-44 to humans. However, the current 3-h scan is too long for human studies, and the kinetics might even be slower in humans than in nonhuman primates. One option is to reduce the scanning time; however, shortening the scan quickly introduced bias and variability (Supplemental Fig. 3). This is not surprising, as the radiotracer clearance time (half-life = ln(2)/*k*_2_) is 175 ± 42 min at baseline (Supplemental Table 1), so the time to equilibrium is long. Another option is to split the scan into early and late phases, giving the subject a rest period. We evaluated the impact of the timing and duration of the rest period on *V*_T_ and *K*_1_ estimates (Supplemental Tables 2 and 3) and found that a 30- or 60-min break starting at 60 or 90 min after radiotracer injection yields minor changes in *V*_T_ and *K*_1_ values.

## CONCLUSION

We successfully developed 4 GAT-1 selective radiotracers and evaluated them in nonhuman primates with kinetic analysis and blocking studies with tiagabine. Of these compounds, ^18^F-GATT-44 exhibited consistent results and reasonable BP_ND_ values and will progress to human studies.

## DISCLOSURE

This work was funded by NIH grant U01MH107803. Its contents are solely the responsibility of the authors and do not necessarily represent the official view of NIH. No other potential conflict of interest relevant to this article was reported.

## Notes

KEY POINTS **QUESTION:** Do the novel GAT-1 radiotracers successfully enter the brain and bind to GAT-1? **PERTINENT FINDINGS:** All 4 GAT-1 novel radiotracers (^18^F-GATT-31, ^18^F-GATT-34, ^18^F-GATT-39, and ^18^F-GATT-44) crossed the BBB, displayed good uptake in the brain and GAT-1 specific binding, which can be blocked with tiagabine and quantified by kinetic analysis using the 1TCM. ^18^F-GATT-44 delivered more consistent overall results, with 69% occupancy, and will progress to human studies. **IMPLICATIONS FOR PATIENT CARE:** In vivo imaging of GAT-1 will provide an important tool to further understand the GABAergic system and its implication in neuropsychiatric disorders.

## References

[1] Conti F Melone M De Biasi S Minelli A Brecha NC Ducati A. Neuronal and glial localization of GAT-1, a high-affinity gamma-aminobutyric acid plasma membrane transporter, in human cerebral cortex: with a note on its distribution in monkey cortex. J Comp Neurol. 1998;396:51–63. 9623887 10.1002/(sici)1096-9861(19980622)396:1<51::aid-cne5>3.0.co;2-h

[2] Conti F Minelli A Melone M. GABA transporters in the mammalian cerebral cortex: localization, development and pathological implications. Brain Res Brain Res Rev. 2004;45:196–212. 15210304 10.1016/j.brainresrev.2004.03.003

[3] Andersen KE Braestrup C Gronwald FC. The synthesis of novel GABA uptake inhibitors. 1. Elucidation of the structure-activity studies leading to the choice of (*R*)-1-[4,4-bis(3-methyl-2-thienyl)-3-butenyl]-3-piperidinecarboxylic acid (tiagabine) as an anticonvulsant drug candidate. J Med Chem. 1993;36:1716–1725. 8510100 10.1021/jm00064a005

[4] Adkins JC Noble S. Tiagabine. A review of its pharmacodynamic and pharmacokinetic properties and therapeutic potential in the management of epilepsy. Drugs. 1998;55:437–460. 9530548 10.2165/00003495-199855030-00013

[5] Eriksson IS Allard P Marcusson J. [^3^H]tiagabine binding to GABA uptake sites in human brain. Brain Res. 1999;851:183–188. 10642842 10.1016/s0006-8993(99)02183-6

[6] Sybirska E Seibyl JP Bremner JD. [^123^I]iomazenil SPECT imaging demonstrates significant benzodiazepine receptor reserve in human and nonhuman primate brain. Neuropharmacology. 1993;32:671–680. 8395663 10.1016/0028-3908(93)90080-m

[7] Motiwala Z Aduri NG Shaye H. Structural basis of GABA reuptake inhibition. Nature. 2022;606:820–826. 35676483 10.1038/s41586-022-04814-x PMC9394549

[8] Fattorini G Melone M Conti F. A reappraisal of GAT-1 localization in neocortex. Front Cell Neurosci. 2020;14:9. 32116556 10.3389/fncel.2020.00009 PMC7031676

[9] Mattison KA Butler KM Inglis GAS. SLC6A1 variants identified in epilepsy patients reduce gamma-aminobutyric acid transport. Epilepsia. 2018;59:e135–e141. 30132828 10.1111/epi.14531

[10] Bitanihirwe BK Woo TU. Transcriptional dysregulation of gamma-aminobutyric acid transporter in parvalbumin-containing inhibitory neurons in the prefrontal cortex in schizophrenia. Psychiatry Res. 2014;220:1155–1159. 25312391 10.1016/j.psychres.2014.09.016 PMC4447488

[11] Schwartz TL Azhar N Husain J. An open-label study of tiagabine as augmentation therapy for anxiety. Ann Clin Psychiatry. 2005;17:167–172. 16433059 10.1080/10401230591002138

[12] Carpenter LL Schecter JM Tyrka AR. Open-label tiagabine monotherapy for major depressive disorder with anxiety. J Clin Psychiatry. 2006;67:66–71. 16426090 10.4088/jcp.v67n0110

[13] Fuhrer TE Palpagama TH Waldvogel HJ. Impaired expression of GABA transporters in the human Alzheimer’s disease hippocampus, subiculum, entorhinal cortex and superior temporal gyrus. Neuroscience. 2017;351:108–118. 28385633 10.1016/j.neuroscience.2017.03.041

[14] Di Palma M Catalano M Serpe C. Lipopolysaccharide augments microglial GABA uptake by increasing GABA transporter-1 trafficking and bestrophin-1 expression. Glia. 2023;71:2527–2540. 37431178 10.1002/glia.24437

[15] Andersson JD Matuskey D Finnema SJ. Positron emission tomography imaging of the gamma-aminobutyric acid system. Neurosci Lett. 2019;691:35–43. 30102960 10.1016/j.neulet.2018.08.010

[16] Murrell E Pham JM Sowa AR. Classics in neuroimaging: development of positron emission tomography tracers for imaging the GABAergic pathway. ACS Chem Neurosci. 2020;11:2039–2044. 32578977 10.1021/acschemneuro.0c00343 PMC7982716

[17] Knippenberg N Bauwens M Schijns O. Visualizing GABA transporters in vivo: an overview of reported radioligands and future directions. EJNMMI Res. 2023;13:42. 37171631 10.1186/s13550-023-00992-5 PMC10182260

[18] Sowa AR Brooks AF Shao X. Development of positron emission tomography radiotracers for the GABA transporter 1. ACS Chem Neurosci. 2018;9:2767–2773. 29763549 10.1021/acschemneuro.8b00183 PMC6249062

[19] Wang C Gravel P Zheng M-Q. Development of novel brain-penetrant radioligands for PET imaging of GABA transporter-1 [abstract]. J Nucl Med. 2021;62(suppl 1):6.33334911

[20] Gu J Gravel P Wang C. Synthesis and evaluation of two novel radioligands for neuroimaging of GABA transporter-1 [abstract]. J Nucl Med. 2022;63(suppl 2):2962.

[21] Percie Du Sert N Hurst V Ahluwalia A. The ARRIVE guidelines 2.0: updated guidelines for reporting animal research. J Cereb Blood Flow Metab. 2020;40:1769–1777. 32663096 10.1177/0271678X20943823 PMC7430098

[22] Hilton J Yokoi F Dannals RF Ravert HT Szabo Z Wong DF. Column-switching HPLC for the analysis of plasma in PET imaging studies. Nucl Med Biol. 2000;27:627–630. 11056380 10.1016/s0969-8051(00)00125-6

[23] Gallezot JD Nabulsi N Neumeister A. Kinetic modeling of the serotonin 5-HT1B receptor radioligand [^11^C]P943 in humans. J Cereb Blood Flow Metab. 2010;30:196–210. 19773803 10.1038/jcbfm.2009.195 PMC2949107

[24] Smart K Zheng MQ Ahmed H. Comparison of three novel radiotracers for GluN2B-containing NMDA receptors in non-human primates: (*R*)-[(11)C]NR2B-Me, (*R*)-[(18)F]of-Me-NB1, and (*S*)-[(18)F]of-NB1. J Cereb Blood Flow Metab. 2022;42:1398–1409. 35209743 10.1177/0271678X221084416 PMC9274863

[25] Hooker JM Carson RE. Human positron emission tomography neuroimaging. Annu Rev Biomed Eng. 2019;21:551–581. 31167104 10.1146/annurev-bioeng-062117-121056

[26] Cunningham VJ Rabiner EA Slifstein M Laruelle M Gunn RN. Measuring drug occupancy in the absence of a reference region: the Lassen plot re-visited. J Cereb Blood Flow Metab. 2010;30:46–50. 19738632 10.1038/jcbfm.2009.190 PMC2949110

[27] Guo Q Owen DR Rabiner EA Turkheimer FE Gunn RN. A graphical method to compare the in vivo binding potential of PET radioligands in the absence of a reference region: application to [(1)(1)C]PBR28 and [(1)(8)F]PBR111 for TSPO imaging. J Cereb Blood Flow Metab. 2014;34:1162–1168. 24736889 10.1038/jcbfm.2014.65 PMC4083379

